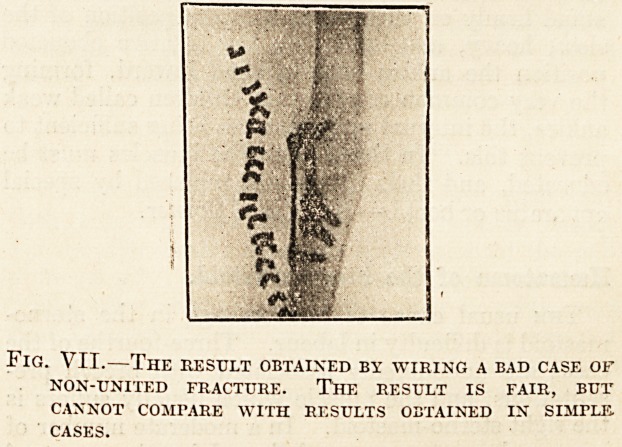# The Operative Treatment of Fractures

**Published:** 1911-01-28

**Authors:** 


### SURGERY.

### THE OPERATIVE TREATMENT OF FRACTURES.

### IV.—UN-UNITED AND MAL-UNITED FRACTURES.

There remains to be discussed the question of operative interference in cases of non-union and mal-union. In the former the operation consists in cutting down upon the fracture, in removing any structures lying in between the broken pieces, in freshening the ends of bone by scraping away the new-formed fibrous tissue, or better, by sawing a thin layer off and then by fixing the bone with plates and screws.

#### Operation in Cases of Mal-union.

In the case of mal-union a similar procedure is gone through, a wedge is removed, or the bone sawn obliquely and extended, or some projecting piece of bone impeding movement is cut off. The technique as regards asepsis is the same as was described under simple fractures. In the case of un-united fractures, however, suppuration is liable to occur even with the most perfect technique, and a well-planned operation result in a disappointing failure. This result does not necessarily depend on the surgeon. When in a series of fracture cases done by the same man and with the same technique cases of un-united fracture suppurate, while every case of operation for simple fracture runs an aseptic course, the cause of suppuration must be sought in some other factor than technique. The cause of the suppuration is probably the same as the original cause of non-union, namely, that the fracture was a compound one, and in that way the organisms were admitted and have lain latent all this time.

#### Operation in Compound Cases.

We have already pointed out that non-union in compound fractures may occur as a result of inflammation without there ever having been any suppuration, and this fact must always be borne in mind when considering an operation for non-union. In considering operation for compound fractures we also strongly advocated that an operation including the insertion of a plate should not be done, and it may well be asked why a similar rule should not also be laid down in regard to those cases which are also liable to suppuration. The answer to this is threefold. First, that we do not advocate operation for delayed union, but only for non-union—that is, we think, measures to obtain union other than operative should be exhausted first. Secondly, that in the fracture when first done the patient has a reasonable chance of a moderately functional limb, whereas the patient with an injured bone has not, and should therefore be prepared to take a greater risk. Thirdly, that the risk is by no means so great in the case of non-union as in the case of a fresh compound fracture. In the latter suppuration after operation is almost the rule, in the former it is a

Fig. VII.—The result obtained by wiring a bad case of non-united fracture. The result is fair, but cannot compare with results obtained in simple cases.

complication the surgeon must bear in mind rather than a condition that he is bound to meet.

#### Non-union in Simple Cases.

We have said that most cases of non-union occur in compound fractures; there remain a few which occur in simple fractures, and which are due to the intervention of soft parts; in these cases there is not the same reason for deferring operation as in the case of the primarily compound, and they may be operated on quite early with good results. Turning to the mal-united fractures, we must here admit that the results are disappointing. When a bone has joined up in bad position there is not the chance of returning the limb to its original position that there was when the fracture was fresh. No doubt some improvement in the junction of the limb may be obtained, but the result should not be compared with the limb before operation, but with what the limb would have been had perfect apposition been obtained in the first instance. We think that this fact does away with the argument against operating on a simple fracture in the first instance, because an operation can always be done later if mal-union results. Nature does not admit of delay; if the surgeon will not do his work in the first instance, she does her best without him, but in doing so she locks the door against his successful intervention later. The above argument should, we maintain, be kept for compound fractures, and even then the operation must be approached only after due consideration, as the risks of asepsis discussed under non-union are present here also. As the improvement in the primary treatment of fractures continues, operations for mal-union will become more and more rare.

We have now discussed the operative treatment of fractures in all its bearings; details of operation for different bones have not been put in. How to approach the bone, and where best to put the plate must be learnt by seeing or by practising these operations.

Mr. Arbuthnot Lane has kindly allowed us to illustrate these articles with photographs of some of his own cases. No. 6 represents a compound fracture operated on by a surgeon who advocates the insertion of an internal splint in these cases. It will be seen that in this case alone has any rarefying osteitis occurred round the metal. This osteitis is sometimes described as occurring in all cases of insertion of a foreign body into a bone; more probably is it dependent upon the accompanying insertion of micro-organisms. By contrasting No. 7 with 6 and the former pictures, our contention laid down in this article is exemplified. No. 7 is the result of an operation for non-union on No. 6. The result is an improvement on No. 6, but cannot be compared with that obtained in the first five cases. This is indeed a case of non-union, but a similar condition is found with mal-union.

## Figures and Tables

**Fig. VII. f1:**